# Electromagnetic noise inhibits radiofrequency radiation-induced DNA damage and reactive oxygen species increase in human lens epithelial cells

**Published:** 2008-05-19

**Authors:** Ke Yao, Wei Wu, KaiJun Wang, Shuang Ni, PanPan Ye, YiBo Yu, Juan Ye, LiXia Sun

**Affiliations:** Eye Center, Affiliated Second Hospital, College of Medicine, Zhejiang University, Hangzhou,China.

## Abstract

**Purpose:**

The goal of this study was to investigate whether superposing of electromagnetic noise could block or attenuate DNA damage and intracellular reactive oxygen species (ROS) increase of cultured human lens epithelial cells (HLECs) induced by acute exposure to 1.8 GHz radiofrequency field (RF) of the Global System for Mobile Communications (GSM).

**Methods:**

An sXc-1800 RF exposure system was used to produce a GSM signal at 1.8 GHz (217 Hz amplitude-modulated) with the specific absorption rate (SAR) of 1, 2, 3, and 4 W/kg. After 2 h of intermittent exposure, the ROS level was assessed by the fluorescent probe, 2',7'-dichlorodihydrofluorescein diacetate (DCFH-DA). DNA damage to HLECs was examined by alkaline comet assay and the phosphorylated form of histone variant H2AX (γH2AX) foci formation assay.

**Results:**

After exposure to 1.8 GHz RF for 2 h, HLECs exhibited significant intracellular ROS increase in the 2, 3, and 4 W/kg groups. RF radiation at the SAR of 3 W/kg and 4 W/kg could induce significant DNA damage, examined by alkaline comet assay, which was used to detect mainly single strand breaks (SSBs), while no statistical difference in double strand breaks (DSBs), evaluated by γH2AX foci, was found between RF exposure (SAR: 3 and 4 W/kg) and sham exposure groups. When RF was superposed with 2 μT electromagnetic noise could block RF-induced ROS increase and DNA damage.

**Conclusions:**

DNA damage induced by 1.8 GHz radiofrequency field for 2 h, which was mainly SSBs, may be associated with the increased ROS production. Electromagnetic noise could block RF-induced ROS formation and DNA damage.

## Introduction

There has been growing concerns about the potential effects of radiofrequency electromagnetic fields (RF EMFs; 10 MHz–300 GHz) on human health, especially the influence on DNA damage, because of wide use of mobile phones, microwave ovens, and so on. The proximal distance of mobile phone to the head has raised anxieties about the biologic effects of microwave radiation (300 MHz–300 GHz) on eyes [1,2].

In recent years, although special attentions have been paid to DNA damage induced by radiofrequency electromagnetic radiation (RFR), the inconsistent results cause controversies. Lai et al. [3,4] reported single-stranded and double-stranded DNA breaks in rat brain cells after 2 h of exposure to 2450 MHz microwaves. DNA damage induced by microwave radiation was also detected by comet assay in other investigations [5,6]. However, Malyapa et al. [7] repeated the research performed by Lai et al. [3,4], but did not confirm the observation of DNA damage after exposure to 2450 MHz microwaves. Other negative results were reported in the literature [8-11].

Oxygen free radicals may play a role in mechanisms of the biologic effects induced by electromagnetic radiation [12,13]. In aerobic cells, reactive oxygen species (ROS) are generated as a by-product of normal mitochondrial activity. If not properly controlled, ROS can cause severe damage to cellular macromolecules, especially DNA [14]. There may be some associations between the overproduction of ROS and DNA damage induced by electromagnetic radiation.

In our previous study, we detected that after 24 h of intermittent exposure (5 min fields on/10 min fields off) to 1.8 GHz radiofrequency field of the Global System for Mobile Communications (GSM) used in mobile phones could induce significant DNA damage and ROS increase in human lens epithelial cells (HLECs), which could be blocked by superposing a “noise” magnetic field (MF) [15]. In this experiment, we intended to observe whether acute (2 h) exposure to microwaves could induce similar effects on cultured human lens epithelial cells.

The alkaline comet assay is considered a sensitive assay for detecting DNA single strand breaks (SSBs), double strand breaks (DSBs), alkali labile sites (ALS), incomplete excision repair sites, etc., but this assay is especially sensitive to SSBs [16]. The immunocytochemical assay of phosphorylated form of H2AX (γH2AX) is an early and specific indicator for the existence of a DSB [17]. We used the two methods to observe DNA damage.

## Methods

### Cell culture

The HLECs, SRA01/04, were obtained from Riken Cell Bank (Tsukuba, Japan) and cultured in Dulbecco’s modified Eagle’s medium (DMEM; Gibco, Grand Island, NY) supplemented with 20% heat-inactivated fetal bovine serum (HIFBS, Hyclone Laboratories Inc., Logan, UT) at 37 °C in a humidified atmosphere of 5% CO_2_. The cells were seeded in a 35-mm dish (NUNC, Roskilde, Denmark) in a total volume of 2 ml. The cells were divided into four groups: sham exposure group, microwave radiation group at the specific absorption rate (SAR) of 1, 2, 3, or 4 W/kg, noise MF group at 2 μT, and microwave radiation group superposed with noise MF for 2 h. The SAR at which energy is absorbed in body tissues (watt per kilogram; W/kg) has been widely adopted at frequencies above ~100 kHz.

### Exposure systems

The exposure system named “sXc-1800 system” that employed a GSM signal was designed by the Foundation for Information Technologies in Society (IT’IS, Zurich, Switzerland). It mainly consists of an RF generator, an arbitrary function generator, a narrow band amplifier, and two rectangular waveguides operating at a frequency of 1.8 GHz. The two waveguides, one for exposure and the other for sham exposure, are placed inside a conventional incubator to ensure constant environmental conditions (37 °C, 5% CO_2_/95% air atmosphere). The increased temperatures of the cells within the culture dish exposed to the SAR of 1, 2, 3, and 4 W/kg were 0.027, 0.054, 0.081, and 0.108 °C, respectively. A dish holder inside the waveguide guarantees that the dishes are placed exactly in the H-field maximum of the standing wave and exposed simultaneously in E polarization inside a waveguide. The system enables the exposure of a monolayer of cells with less than a 30% non-uniformity of specific absorption rate (SAR). Six Petri dishes can be exposed simultaneously in one exposure waveguide. The entire setup is computer controlled, enabling automated control of the exposure parameters including exposure strength (SAR), exposure time, and exposure pattern. The RF EMF simulating the GSM 1.8 GHz signal is amplitude-modulated by a rectangular pulse with a repetition frequency of 217 Hz and a duty cycle of 1:8. The HLECs were intermittently (5 min fields on/10 min fields off) exposed or sham-exposed to RF EMF for 2 h at an average SAR of 1, 2, 3, or 4 W/kg.

To generate a noise MF, both sides of the waveguides of the sXc-1800 system were wrapped with two rectangular Helmholtz coils. The center distance of two Helmholtz coils is 24 cm. The direction of the coils is the same as the circular wires in the RF waveguides, and the direction of the noise MF is consistent with the magnetic field of microwave radiation. The coils were provided with a 30–90 Hz white noise signal (generated through software designed by Dr. Penafiel, Catholic University of America, Washington, DC). The amplitude of the noise MF was 2 μT in the experiment.

### Intracellular reactive oxygen species detection

The 2',7'-dichlorodihydrofluorescein diacetate (DCFH-DA) method was used to detect ROS production [18]. DCFH-DA enters cells and is further oxidized by ROS with the formation of a fluorescent product (DCF). Cells were incubated for 30 min at 37 °C with DCFH-DA solution with a final concentration of 50 µM. After incubation, cells were rinsed twice with PBS and collected with trypsin-EDTA solution (0.25% trypsin-0.02% ethylenediamine tetraacetic acid solution; Gibco, Grand Island, NY). After centrifugation at 1500 rpm for 5 min, the supernatant was discarded. The pellet was suspended in PBS. Fluorescence of the samples was monitored at an excitation wavelength of 485 nm and an emission wavelength of 538 nm. The ROS level was expressed as OD/mg protein. Protein concentration was determined using the Bradford method. ROS production in exposed samples was expressed as a percentage of the sham-exposed ones.

### Comet assay

The alkaline (pH>13) single cell gel electrophoresis (SCGE) assay was essentially performed according to the description given by Singh et al. [19]. 0.65% Normal melting agarose (NMA; 0.65%) and 0.65% low melting agarose (LMA) was prepared in Ca^2+^, Mg^2+^ free PBS. Cells were suspended in LMA, and 75 μl of the LMA-cell suspension was piped onto a frosted glass microscope slide pre-coated with a 100 μl layer of 0.65% NMA. The third layer of 75 μl of 0.65% LMA was subsequently added. Then, the slides were immersed in freshly prepared ice-cold lysis solution (1% N-lauroylsarcosine sodium salt, 2.5 M NaCl, 100 mM Na_2_ EDTA, 10 mM Tris–HCl, 1% Triton X-100, and 10% DMSO, pH=10) to lyse cell proteins and allow DNA unfolding. After at least 1 h in the dark at 4 °C, the slides were covered with fresh buffer (1 mM Na_2_ EDTA, 300 mM NaOH, pH>13) in a horizontal electrophoresis unit. The slides were left in this buffer for 20 min to allow DNA unwinding. The DNA was then electrophoresed at 20 V and 300 mA for 20 min. Both unwinding and electrophoresis were performed at 4 °C. The slides were washed gently in a neutralization buffer (0.4 M Tris–HCl, pH=7.5) to remove alkali and detergent and fixed in methanol for 3 min, then stained with 50 μl ethidium bromide (20 μg/ml). All of the steps described above were conducted under yellow light or in the dark to prevent additional DNA damage. Pictures were taken individually at 400X magnification using fluorescent microscopy (Olympus BX51; Olympus Optical Co., LTD., Tokyo, Japan) equipped with a 530 nm excitation filter, a 590 nm emission filter, and a digital camera (Olympus DP50; Olympus Optical Co.). Nuclear width and the extent of migration of DNA fragments, the mean tail length (MTL), and the mean tail moment (MTM) were analyzed using the Image-Pro Plus program (Media Cybernetics Inc., Bethesda, MD).

### Immunofluorescent microscope detection of γH2AX foci

HLECs were fixed in 4% paraformaldehyde for 15 min, washed with PBS, and permeabilized in 0.2% Triton X-100. Goat blocking serum (Beijing Zhongshan Biotechnology Co., Beijing, China) was used to block nonspecific binding at 25 °C for 2 h. HLECs were incubated with a 1:1000 mouse monoclonal anti-γH2AX antibody (Upstate Technology, Lake Placid, NY) for 2 h followed with 1:500 FITC-conjugated goat anti-mouse IgG secondary antibody (Beijing Zhongshan Biotechnology Co.) for 1 h. To stain nuclei, DAPI was added to the cells and incubated for another 15 min. The coverslip was then removed from the plate and mounted onto a glass slide and observed with an Olympus AX70 fluorescent microscope (Olympus). Image Pro Plus (Media Cybernetics Inc.) was used to count the γH2AX foci in each cell. The positive control was exposed to the chemical mutagen, 4-nitroquinoline-1-oxide (4NQO) at 0.01 μmol/l for 1 h.

### Statistical analysis

Data were expressed as mean±SD from three independent experiments and analyzed with one-way ANOVA followed by a post hoc application of Dunnett’s test. A p value of less than 0.05 was considered statistically significant.

## Results

### Reactive oxygen species increase induced by microwave radiation

**Figure 1:**
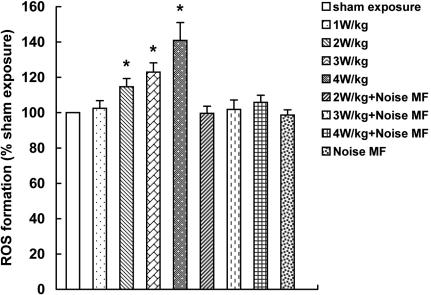
Reactive oxygen species levels in HLECs after 1.8 GHz microwave treatment with or without superposing with noise MF. Intracellular ROS significantly increased after 2 h of exposure at the SAR of 2, 3, and 4 W/kg, which was suppressed when superposed with electromagnetic noise. The asterisk indicates that p<0.05.

Figure 1 shows that intracellular ROS significantly increased after 2 h of exposure to a 1.8 GHz radiofrequency field at the SAR of 2, 3, and 4 W/kg (p<0.05), which were suppressed when superposed with electromagnetic noise (p>0.05). No statistical elevation of ROS level was detected in the 1 W/kg group (p>0.05). Electromagnetic noise alone had no significant influence on intracellular ROS formation compared with sham exposure control (p>0.05).

### DNA damage detected by comet assay after exposure to microwaves

**Figure 2:**
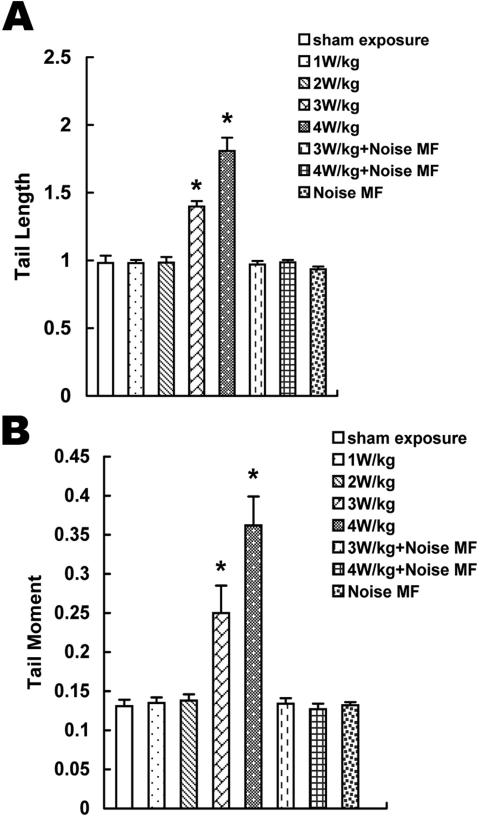
The results of alkaline comet assay after 1.8 GHz microwave treatment with or without superposing with noise MF. **A**: The mean tail length (MTL). **B**: The mean tail moment (MTM). The MTL and MTM of comet assay induced by 1.8 GHz microwave radiation at the SAR of 3 W/kg and 4 W/kg was significantly higher than sham exposure whereas no significant differences could be observed in other exposure groups compared with the sham exposure group. The significant DNA damage induced by 3 W/kg and 4 W/kg microwave radiation was blocked by superposing with electromagnetic noise. The asterisk indicates that p<0.001.

The MTL and MTM results of the alkaline comet assay were shown in Figure 2.

DNA damage induced by 1.8 GHz microwave radiation at the SAR of 3 W/kg and 4 W/kg was significantly higher than sham exposure (p<0.001) whereas no significant differences could be observed in other exposure groups compared with the sham exposure group (p>0.05). Electromagnetic noise alone did not increase DNA damage of HLEC, and when it was superposed on the radiofrequency field, the electromagnetic noise could block RF-induced DNA damage.

### No significant changes of double strand breaks after microwave radiation detected by γH2AX foci formation test

**Figure 3:**
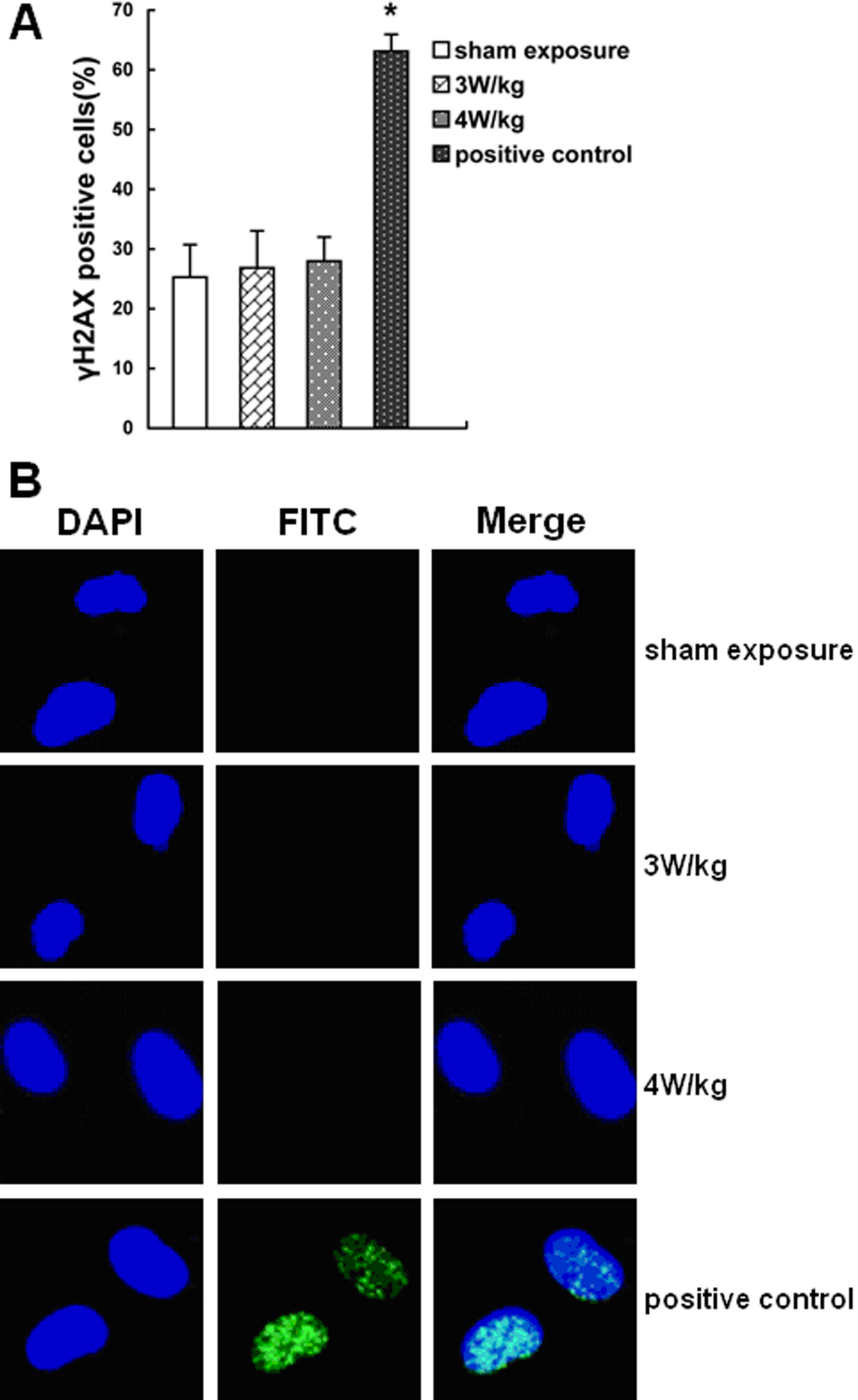
The results of γH2AX foci formation assay after 1.8 GHz microwave treatment. **A**: The percentage of γH2AX positive cells is shown. No significant changes of DSBs were detected after exposure to the 3 W/kg and 4 W/kg radiation. **B**: Images of γH2AX foci is shown. The nuclei stained by DAPI exhibit in blue, while the DSBs stained by FITC exhibit in green. The asterisk indicates that p<0.001

The γH2AX foci were used as an indicator for DSB formation within the nucleus (Figure 3). The percentages of γH2AX foci positive cells in 3 W/kg and 4 W/kg groups were 26.85±6.19% and 27.97±4.05%, respectively, neither of which was significantly different compared with the sham exposure group (25.29±5.44%; p>0.05). The DSBs were higher in the positive control group treated by chemical mutagen, 4NQO, (63.1±2.85%) than in the sham exposure group (p<0.001).

## Discussion

Cataract is one of the major causes of blindness throughout the world. The lens is sensitive to electromagnetic radiation since it is non-vascularized and non-innervated and contains a high percentage of water. The lens epithelial cell plays an important role in maintaining the metabolic homeostasis and transparency of the entire lens and its dysfunction can be an early event in cataractogenesis. Both oxidative stress and DNA damage of lens epithelial cells can result in opacification of the lens [20-22].

The alkaline comet assay (pH>13) is sensitive for detecting DNA damage, especially for SSBs. Previously, we have reported that 1.8 GHz RF EMF (SAR: 3 W/kg) radiation could induce repairable DNA damage in HLECs after 2 h of continuous exposure [23]. In this study, 2 h of intermittent exposure to microwaves at the SAR of 3 W/kg and 4 W/kg could induce significant DNA damage, was evaluated by alkaline comet assay. This result was consistent with the effects of long time exposure (24 h) that we reported previously [15]. On the other hand, the assay for DSBs show that there was no significant difference between exposed (SAR: 3 and 4 W/kg) and sham-exposed groups. Since it has been reported that DSBs induce the phosphorylation of histone variant H2AX at serine 139 (γH2AX), an immunocytochemical assay with antibodies recognizing γH2AX has become the gold standard for detection of DSBs [17,24-27]. We proposed that DNA damage induced by acute exposure (2 h) to RFR may be mainly SSBs. Since 24 h exposure to microwaves at the SAR of 4 W/kg could induce significant DSBs in the same cells [15], we supposed the effects of microwave radiation on DNA damage were associated with exposure time.

It has been reported that mobile phones could induce oxidative stress in corneal and lens tissues [28]. ROS, which include superoxide, hydrogen peroxide, hydroxyl radicals, singlet oxygen, and so on, can cause several types of DNA damage such as oxidized bases and single-strand and double-strand breaks. DNA damage produced by ROS is the most frequently occurring damage [29]. In the present study, intracellular ROS of HLECs were increased significantly after 2 h of exposure to microwaves at the SAR of 2, 3, and 4 W/kg compared to sham-exposed HLECs. Previously, we detected an ROS increase after microwave radiation for 24 h in 3 and 4 W/kg exposed groups but not in the 2 W/kg group [15]. It is possible that the overproduction of ROS induced by acute exposure to 2 W/kg were later scavenged by protection mechanisms such as antioxidants and antioxidant enzymes of lens epithelial cells.

How does the electromagnetic field affect the biologic system? Litovitz et al. [30-33] proposed that living cells exist in an electrically noisy environment and these endogenous thermal noise fields are larger than those exogenous EMFs reported to cause effects. They suggested that only the EMFs that are temporally and spatially coherent such as radiofrequency fields could affect living cells while endogenous thermal noise fields, which cells do not respond to, were temporally and spatially incoherent. Coherence was an essential character for EMFs to cause bioeffects, which means the characteristic parameters of EMFs are constant over a period of time (>~10 s) [30-34]. It was speculated that when an incoherent random noise field is superimposed on a coherent EMF signal, any observed EMF-induced bioeffects would be suppressed. A few observations [34-36] have supported this theory. In this experiment, the cellular effects induced by acute microwave radiation were mitigated by superposing with electromagnetic noise in vitro. Further investigation on the temporal-and-spatial coherency hypothesis is required on different cell types and different doses as well as on modulations of RF EMFs.
